# Dynamic Changes in Peripheral Nerve Stiffness After Regional Anesthesia: Implications of Shear Wave Elastography in Adductor Canal Block

**DOI:** 10.3390/jcm15135306

**Published:** 2026-07-07

**Authors:** Hyeonsook Jee, Sung-woo Hyung, Yuseung Oh, Hye Joo Yun

**Affiliations:** 1 Department of Anesthesiology and Pain Medicine, Eunpyeong St. Mary’s Hospital, College of Medicine, The Catholic University of Korea, Seoul 03312, Republic of Korea; 2 Department of Anesthesiology and Pain Medicine, Yeouido St. Mary’s Hospital, College of Medicine, The Catholic University of Korea, Seoul 07345, Republic of Korea

**Keywords:** adductor canal block, shear wave elastography, peripheral nerve stiffness, saphenous nerve, regional anesthesia

## Abstract

Adductor canal block (ACB) is widely used for perioperative analgesia in knee surgery because it provides effective pain control while preserving quadriceps muscle strength. With the increasing use of ultrasound-guided regional anesthesia, interest has expanded beyond conventional morphologic imaging toward quantitative assessment of peripheral nerve function and biomechanics. Shear wave elastography (SWE) is an emerging ultrasound-based technique that enables real-time quantification of tissue stiffness and has recently gained attention in peripheral nerve evaluation. Previous SWE studies have primarily focused on chronic neuropathic conditions, including entrapment neuropathy and diabetic neuropathy, in which increased nerve stiffness is commonly observed. However, emerging observations suggest that peripheral nerve stiffness may dynamically decrease following regional anesthesia procedures such as ACB. This finding raises the possibility that nerve stiffness reflects not only chronic structural pathology but also transient physiologic and biomechanical modulation. Potential mechanisms underlying reduced stiffness after ACB include perineural hydrodissection, decreased fascial compression, sympathetic blockade-induced vasodilation, altered intraneural pressure, and changes in surrounding muscle tension. Because the saphenous nerve within the adductor canal is superficial and consistently visualized under ultrasound guidance, ACB represents an attractive model for investigating dynamic changes in peripheral nerve biomechanics. This narrative review summarizes current evidence regarding SWE assessment of peripheral nerves and discusses the potential implications of dynamic stiffness changes after regional anesthesia. We review the biomechanical principles of SWE, factors affecting nerve stiffness, current evidence in neuropathic and perioperative settings, technical limitations, and future clinical applications. Understanding the dynamic behavior of peripheral nerve stiffness may expand the role of SWE from a diagnostic tool for neuropathy to a quantitative biomarker for regional anesthesia and perioperative nerve physiology.

## 1. Introduction

Adductor canal block (ACB) has become one of the most widely used regional analgesic techniques for knee surgery because it provides effective postoperative analgesia while relatively preserving quadriceps muscle strength. Compared with femoral nerve block, ACB offers the advantage of improved early mobilization and reduced risk of postoperative falls, leading to its increasing adoption in enhanced recovery after surgery protocols [1,2,3]. Despite its widespread clinical use, the physiologic and biomechanical effects of ACB on peripheral nerves and surrounding tissues remain incompletely understood.

Ultrasound guidance has substantially improved the precision and safety of regional anesthesia. Conventional B-mode ultrasonography primarily provides morphologic information, including nerve size, fascial anatomy, and local anesthetic spread. However, structural imaging alone may not fully reflect dynamic physiologic changes occurring within peripheral nerves after local anesthetic injection [4]. In recent years, shear wave elastography (SWE) has emerged as a promising ultrasound-based imaging modality capable of quantitatively assessing tissue stiffness in real time. SWE measures the propagation velocity of mechanically induced shear waves through tissues, allowing indirect estimation of tissue elasticity [5,6].

Peripheral nerve stiffness has gained attention as a potential biomarker for neural health and pathology. Increased nerve stiffness has been reported in several entrapment neuropathies, diabetic neuropathy, and inflammatory nerve disorders [7,8,9,10]. Changes in neural stiffness may reflect alterations in intraneural edema, fibrosis, vascular congestion, or mechanical compression. Consequently, quantitative stiffness assessment using SWE may provide additional functional information beyond conventional sonographic morphology [11].

Within the field of regional anesthesia, the interaction between perineural injection and neural biomechanics remains poorly characterized. Local anesthetic injection may transiently alter intraneural pressure, fascial compartment compliance, and surrounding tissue tension, potentially influencing nerve stiffness measurements [12,13]. Understanding these mechanical responses may improve insight into block dynamics, injection safety, and early detection of neural injury. Nevertheless, only limited studies have investigated shear wave elastographic changes associated with peripheral nerve blocks [14,15]. Most published SWE research has focused on peripheral neuropathies or normative nerve stiffness values, whereas dynamic alterations in nerve mechanical properties following regional anesthesia remain poorly characterized.

The adductor canal represents an especially attractive model for investigating peripheral nerve stiffness during regional anesthesia. The saphenous nerve is relatively superficial, consistently identifiable under ultrasound guidance, and located within a confined fascial compartment that may demonstrate measurable biomechanical changes following injectate administration. Furthermore, the growing use of continuous catheter-based ACB techniques provides opportunities for serial stiffness assessment over time [16,17].

This narrative review aims to summarize current knowledge regarding peripheral nerve stiffness assessment using shear wave elastography, with particular focus on its potential applications in adductor canal block. We discuss the biomechanical principles of SWE, factors affecting nerve stiffness, current evidence related to peripheral nerve blocks, technical limitations of elastographic assessment, and future clinical implications for regional anesthesia practice.

## 2. Principles of Shear Wave Elastography

### 2.1. Physical Principles of Shear Wave Elastography

Shear wave elastography (SWE) is an ultrasound-based imaging modality that quantitatively evaluates tissue stiffness by measuring the propagation velocity of mechanically induced shear waves through biological tissues. Unlike conventional B-mode ultrasonography, which primarily provides structural and morphologic information, SWE enables functional assessment of tissue mechanical properties in real time [5,6]. In SWE, acoustic radiation force impulses generated by the ultrasound transducer induce localized tissue displacement. This displacement creates transverse shear waves that propagate perpendicular to the direction of the ultrasound beam. The velocity of shear wave propagation is directly related to tissue stiffness; stiffer tissues transmit shear waves more rapidly than softer tissues [18].

(1) $$v_{s}=\sqrt{\begin{array}{c} \frac{G}{\begin{array}{c} \rho \end{array}} \end{array}}$$

In this equation, $v_{s}$ represents shear wave velocity, G denotes shear modulus, and ρ indicates tissue density. Because tissue density remains relatively constant in most soft tissues, shear wave velocity primarily reflects differences in tissue elasticity [19].

Many ultrasound systems additionally estimate Young’s modulus using the relationship:

(2) E≈3G

where E represents Young’s modulus and G is the shear modulus, assuming tissue isotropy and incompressibility. Measurements are typically expressed either in meters per second (m/s) or kilopascals (kPa). However, peripheral nerves exhibit anisotropic and heterogeneous mechanical properties, which may limit the accuracy of simplified elastic models [20].

**Figure 1:**
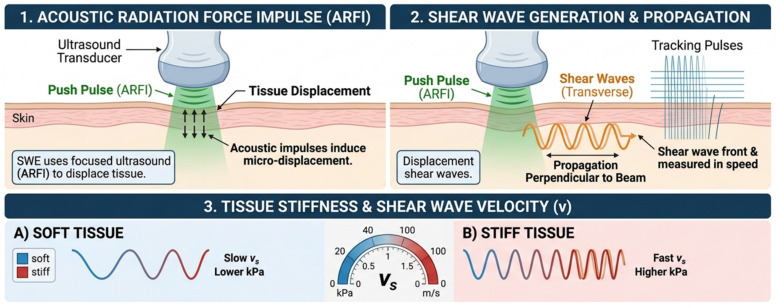
Schematic diagram of the fundamental principles and equations of Shear Wave Elastography (SWE). Figure created using Gemini (https://gemini.google.com), accessed on 15 May 2026.

Compared with strain elastography, SWE offers several advantages for peripheral nerve assessment. SWE is less operator-dependent because tissue deformation is generated automatically by acoustic impulses rather than manual probe compression. In addition, quantitative stiffness values improve reproducibility and facilitate longitudinal comparisons (Figure 1) [21].

### 2.2. Shear Wave Elastography in Peripheral Nerves

Application of SWE to peripheral nerves has expanded considerably during the past decade. Peripheral nerves possess unique structural characteristics that influence elastographic measurements. The fascicular arrangement of nerve fibers, surrounding connective tissue layers, and longitudinal fiber orientation create highly anisotropic mechanical behavior [10]. Consequently, stiffness values vary substantially according to probe orientation relative to the nerve axis.

Longitudinal imaging generally produces higher stiffness measurements than transverse imaging because shear waves propagate differently along aligned fascicular structures [8]. For this reason, standardization of imaging planes is essential for reliable comparison among studies. Probe pressure also significantly affects measured stiffness values, as even minimal transducer compression may artificially increase nerve stiffness [22].

Several studies have demonstrated the feasibility of SWE in evaluating normal peripheral nerves, including the median, tibial, sciatic, and ulnar nerves [7,9,23,24]. In healthy individuals, peripheral nerves typically demonstrate lower stiffness than tendons but higher stiffness than surrounding adipose tissue [25].

Peripheral nerve stiffness is also influenced by dynamic physiologic conditions. Limb position, joint angle, and surrounding muscle contraction can alter nerve tension and excursion, thereby affecting elastographic measurements [26]. For example, extension of adjacent joints may increase neural strain and stiffness, whereas relaxation of surrounding tissues may reduce stiffness. These dynamic properties are particularly relevant in regional anesthesia research because local anesthetic injection may transiently modify compartmental tension and neural biomechanics [26,27].

### 2.3. Technical Considerations and Limitations

Although SWE provides promising quantitative information, several technical limitations must be considered when interpreting peripheral nerve stiffness measurements. One of the most important challenges is the anisotropic nature of neural tissue. Since peripheral nerves are composed of longitudinally organized fascicles, stiffness measurements may vary substantially according to insonation angle and probe orientation [28].

Depth-related attenuation is another important limitation. Shear wave signals weaken as tissue depth increases, potentially reducing measurement reliability in deep structures. Fortunately, the saphenous nerve within the adductor canal is relatively superficial, making it well suited for elastographic evaluation [29].

Region-of-interest (ROI) selection also influences measurement reproducibility. Small ROIs may inadequately represent heterogeneous neural tissue, whereas excessively large ROIs may include surrounding fascia or vascular structures. Additionally, motion artifacts caused by patient movement, vascular pulsation, or muscle contraction can interfere with accurate shear wave propagation analysis [30].

Inter-device variability remains a major obstacle to widespread clinical standardization. Different ultrasound manufacturers employ distinct algorithms, transducer frequencies, and postprocessing techniques, which may produce non-comparable stiffness values. Consequently, absolute cutoff values for normal and abnormal nerve stiffness have not yet been universally established [31].

Another limitation is the uncertain biologic interpretation of stiffness measurements. Increased stiffness may reflect fibrosis, edema, inflammation, compression, or altered vascularity, while decreased stiffness may result from reduced tissue tension, hydrodissection, or changes in neural loading conditions. Therefore, elastographic findings should be interpreted within the broader physiologic and clinical context.

### 2.4. Implications for Regional Anesthesia Research

The ability of SWE to quantitatively assess dynamic tissue mechanics has important implications for regional anesthesia. Conventional ultrasonography primarily evaluates needle position and local anesthetic spread, whereas SWE may provide additional information regarding tissue response following injection [4].

Emerging evidence suggests that peripheral nerve stiffness may change acutely after regional anesthesia procedures [14]. These alterations may reflect mechanical decompression, fascial expansion, altered intraneural pressure, or sympathetic blockade-induced vascular changes. Unlike chronic neuropathic disorders characterized by stiffness elevation, regional anesthesia may produce transient decreases in stiffness associated with physiologic modulation rather than structural injury [9,26].

Because the adductor canal contains a superficial and reproducibly visualized sensory nerve within a confined fascial environment, ACB represents an attractive experimental model for studying these dynamic responses. Serial SWE measurements before and after block performance may improve understanding of injectate biomechanics, compartmental compliance, and nerve physiology during regional anesthesia.

Future investigations integrating SWE with injection pressure monitoring, nerve perfusion imaging, and clinical analgesic outcomes may further clarify the role of elastography as a quantitative biomarker in regional anesthesia practice.

## 3. Peripheral Nerve Stiffness in Health and Disease

### 3.1. Normal Peripheral Nerve Elasticity

Peripheral nerves are mechanically dynamic structures that continuously adapt to physiologic movement and changes in surrounding tissue tension. Under normal conditions, peripheral nerves exhibit substantial elasticity and compliance, allowing them to tolerate elongation, compression, and excursion during limb motion without structural injury [27,32,33]. This biomechanical adaptability is primarily determined by the organization of nerve fascicles and connective tissue components, including the epineurium, perineurium, and endoneurium [33].

The mechanical behavior of peripheral nerves is viscoelastic rather than purely elastic. Consequently, nerve deformation depends not only on the magnitude of external force but also on the duration and rate of applied stress [33]. In vivo studies have demonstrated that peripheral nerves undergo longitudinal excursion and strain during normal joint movement. For example, limb extension may increase neural tension and transiently elevate stiffness measurements on shear wave elastography (SWE) [27].

Normal stiffness values vary considerably among different peripheral nerves because of differences in fascicular composition, surrounding soft tissue, depth, and anatomic location. In addition, physiologic factors such as age, body habitus, limb position, vascular perfusion, and surrounding muscle activity may influence elastographic measurements [34]. Despite increasing interest in SWE, universally accepted reference ranges for normal peripheral nerve stiffness have not yet been established.

Several studies have demonstrated that healthy peripheral nerves generally exhibit relatively homogeneous elastographic patterns with moderate stiffness values [11]. Compared with tendons, nerves are typically softer because of their higher water content and less densely organized collagen structure [25,33]. However, substantial heterogeneity exists among studies owing to differences in ultrasound systems, acquisition protocols, probe orientation, and region-of-interest selection.

Importantly, peripheral nerve stiffness should not be regarded as a static mechanical property. Instead, stiffness likely reflects a dynamic interaction among neural tissue composition, intraneural pressure, vascular perfusion, surrounding fascial tension, and external mechanical loading conditions [14,33,34,35]. This concept is particularly relevant in perioperative settings, where regional anesthesia and local tissue manipulation may acutely alter neural biomechanics.

### 3.2. Peripheral Nerve Stiffness in Entrapment Neuropathy

Entrapment neuropathy is the most extensively studied clinical application of SWE in peripheral nerve imaging. Chronic compression of peripheral nerves produces structural and physiologic alterations including edema, fibrosis, ischemia, demyelination, and impaired axonal transport [36]. These pathologic changes are believed to increase neural stiffness and thereby alter elastographic measurements.

Carpal tunnel syndrome (CTS) has been the most frequently investigated condition in SWE research. Multiple studies have demonstrated significantly increased median nerve stiffness in patients with CTS compared with healthy controls [7,23]. Elevated stiffness has been associated with increased intraneural pressure, vascular congestion, connective tissue proliferation, and chronic mechanical compression within the carpal tunnel.

Similarly, increased stiffness has been observed in ulnar neuropathy at the elbow and tarsal tunnel syndrome [8,11]. In many cases, elastographic changes correlate with symptom severity and electrodiagnostic abnormalities, suggesting that SWE may provide complementary diagnostic information beyond conventional ultrasonography. However, variability in cutoff values and measurement techniques has limited the establishment of standardized diagnostic criteria.

The pathophysiology of stiffness elevation in entrapment neuropathy appears multifactorial. Chronic compression may impair venous outflow and intraneural microcirculation, resulting in edema and inflammatory changes. Over time, repetitive mechanical stress may lead to fibrosis and thickening of connective tissue layers, thereby increasing overall nerve rigidity [22].

These findings have contributed to the prevailing assumption that increased peripheral nerve stiffness primarily reflects pathologic change. However, this paradigm may incompletely represent the dynamic nature of neural biomechanics. Acute alterations in tissue tension and the surrounding mechanical environment may also significantly affect stiffness measurements independent of structural injury.

### 3.3. Peripheral Nerve Stiffness in Metabolic and Inflammatory Neuropathy

Beyond entrapment neuropathy, SWE has also been investigated in systemic neuropathic disorders such as diabetic peripheral neuropathy. Several studies have reported increased stiffness of the tibial nerve and other peripheral nerves in diabetic patients, even in the absence of overt clinical symptoms [9,37,38]. These findings suggest that elastographic abnormalities may precede conventional electrophysiologic changes.

Potential mechanisms underlying increased nerve stiffness in diabetic neuropathy include chronic hyperglycemia-induced connective tissue remodeling, microvascular dysfunction, accumulation of advanced glycation end products, and intraneural edema [39]. These pathophysiologic processes may promote fibrosis, collagen deposition, and structural alteration of neural connective tissue components, thereby reducing neural compliance and impairing normal physiologic mobility. Increased stiffness detected by shear wave elastography may therefore reflect both metabolic and structural changes occurring within the diabetic nerve [9,40].

Inflammatory neuropathies may also alter nerve stiffness. Experimental and clinical studies suggest that inflammation-related edema and cellular infiltration can modify neural elasticity, although reported SWE findings have been inconsistent. Some inflammatory conditions demonstrate increased stiffness due to tissue swelling and fibrosis, whereas acute inflammatory edema may occasionally produce transient softening depending on the balance between fluid accumulation and connective tissue tension [41].

Peripheral nerve trauma may induce dynamic alterations in mechanical properties throughout the healing process. Acute injury is commonly associated with inflammation, vascular changes, and intraneural edema, which may modify tissue compliance and elastographic characteristics. During later stages of healing, scar formation, fibrosis, and connective tissue remodeling can alter the local mechanical environment and contribute to increased neural stiffness [42]. Consequently, elastographic findings after nerve injury may vary according to injury severity, chronicity, and the extent of neural regeneration and tissue remodeling.

Collectively, these observations indicate that peripheral nerve stiffness reflects a complex interaction between structural pathology and physiologic state. Although chronic neuropathic conditions frequently demonstrate stiffness elevation, elastographic measurements are not exclusively markers of irreversible tissue injury. The characteristics of peripheral nerve stiffness in normal and pathologic conditions are summarized in Table 1.

### 3.4. Dynamic Nature of Peripheral Nerve Stiffness

Recent advances in elastographic imaging have increasingly challenged the traditional concept of peripheral nerve stiffness as a fixed mechanical parameter. Experimental and in vivo studies have demonstrated that nerve stiffness is highly responsive to changes in neural loading, limb position, joint movement, and surrounding tissue tension. These findings suggest that elastographic stiffness reflects a dynamic biomechanical and physiologic state rather than an immutable structural property of the nerve [26,35,43].

This dynamic perspective has important implications for regional anesthesia research. Unlike chronic neuropathic disorders characterized by progressive structural abnormalities, regional anesthesia may induce transient changes in tissue mechanics without permanent injury. Perineural injection, hydrodissection, altered fascial tension, and sympathetic blockade may acutely modify the mechanical environment surrounding peripheral nerves.

Understanding peripheral nerve stiffness as a dynamic and context-dependent parameter may expand the role of SWE beyond diagnosis of neuropathy alone. In perioperative medicine, elastography may eventually serve as a quantitative biomarker for evaluating tissue response to regional anesthesia, monitoring nerve physiology, and improving understanding of neural biomechanics in vivo.

## 4. Regional Anesthesia and Dynamic Changes in Nerve Stiffness

### 4.1. Mechanical Effects of Perineural Injection

Regional anesthesia involves deliberate deposition of local anesthetic solution into tissue planes surrounding peripheral nerves. Although the primary clinical objective is interruption of neural signal transmission, perineural injection simultaneously induces substantial mechanical alterations within the surrounding soft tissue environment [4]. These biomechanical changes may significantly influence peripheral nerve stiffness as measured by shear wave elastography (SWE).

Injection of fluid into confined fascial compartments generates transient increases in local tissue pressure and produces displacement of adjacent connective tissue structures. This process resembles hydrodissection, in which fluid mechanically reduces tissue adhesion and decompresses interfascial planes [44].

Hydrodissection may increase neural mobility and decrease external mechanical constraint on the nerve. Consequently, reduced resistance to shear wave propagation may manifest as decreased elastographic stiffness after block performance. Such findings contrast with chronic neuropathic conditions, in which fibrosis and sustained compression increase tissue rigidity [10].

**Figure 2:**
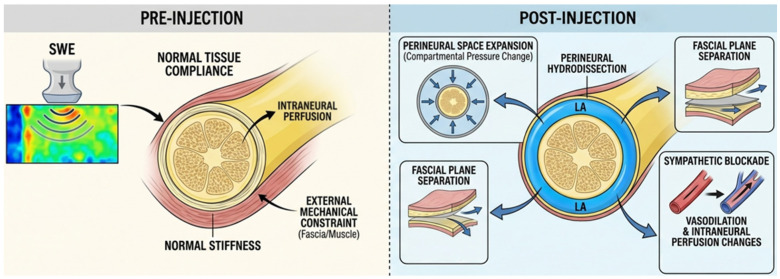
Biomechanical cascade influencing peripheral nerve stiffness after regional anesthesia. Before injection, nerve stiffness reflects the balance between intrinsic neural elasticity, intraneural perfusion, tissue compliance, and external mechanical constraints from the surrounding fascial compartment. Injection of local anesthetic into the confined perineural space transiently elevates local tissue pressure, producing expansion of the perineural space and displacement of adjacent connective tissue structures. This process facilitates hydrodissection and separation of fascial planes, reducing external mechanical loading on the nerve. Simultaneously, sympathetic blockade promotes vasodilation and increases intraneural perfusion, leading to improved microvascular compliance. Together, these mechanical and physiologic changes may explain the decrease in peripheral nerve stiffness observed by shear wave elastography after regional anesthesia. Figure created using Gemini (https://gemini.google.com), accessed on 15 May 2026. Abbreviations: SWE, shear wave elastography.

Perineural injection may additionally modify local stress distribution within the fascial compartment. Expansion of the injectate pocket can redistribute mechanical load away from the nerve and toward surrounding soft tissues, thereby altering neural strain patterns. Because peripheral nerves are highly compliant viscoelastic structures, even subtle changes in external loading conditions may significantly influence stiffness measurements (Figure 2) [45].

The extent of these biomechanical effects likely depends on several procedural variables, including injectate volume, injection pressure, needle position, and fascial compliance. Larger injectate volumes may produce more extensive fascial separation and tissue decompression, whereas elevated injection pressures may transiently increase intraneural stress [12]. Understanding these interactions is important for interpreting elastographic findings after regional anesthesia.

### 4.2. Physiologic Effects of Local Anesthetics on Neural Tissue

In addition to mechanical effects, local anesthetics may induce physiologic changes capable of altering peripheral nerve stiffness. Blockade of sympathetic fibers can produce vasodilation and increased regional blood flow, potentially modifying intraneural vascular dynamics and extracellular fluid distribution [46].

**Figure 3:**
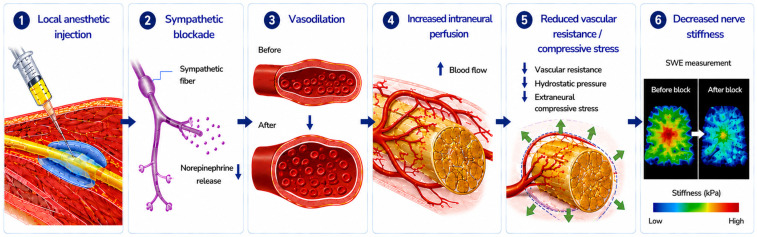
Physiologic mechanisms underlying decreased peripheral nerve stiffness after local anesthetic injection. (1) Local anesthetic is injected near the target nerve (e.g., adductor canal block). (2) The local anesthetic blocks sympathetic efferent fibers, resulting in diminished norepinephrine release. (3) Loss of sympathetic tone causes vascular smooth muscle relaxation and vasodilation. (4) Vasodilation increases blood flow within and around the nerve through the vasa nervorum and endoneurial vessels, leading to enhanced intraneural perfusion. (5) Improved microvascular compliance reduces vascular resistance, hydrostatic pressure, and extraneural compressive stress applied to the nerve. (6) These integrated changes reduce mechanical loading and result in decreased peripheral nerve stiffness, as demonstrated by lower shear wave elastography (SWE) values after the block. Figure created using ChatGPT (https://chatgpt.com), accessed on 20 May 2026.

Changes in intraneural perfusion may influence the mechanical properties of peripheral nerves by altering endoneurial fluid dynamics, hydrostatic pressure, and connective tissue tension within the neural compartment. Experimental studies have demonstrated close interactions between neural microcirculation and intraneural pressure, suggesting that vascular factors contribute to the biomechanical environment of the nerve. Consequently, regional increases in perfusion and changes in sympathetic tone may modify elastographic stiffness through alterations in vascular compliance, tissue hydration, and local mechanical loading conditions (Figure 3) [47,48].

Local anesthetics may also affect surrounding muscular structures. Reduced nociceptive input and altered motor activity may decrease resting muscle tone in adjacent muscles. Since muscular tension contributes to compartmental pressure, relaxation of surrounding tissues may indirectly reduce compressive forces applied to the nerve [49].

Furthermore, perineural injection may transiently modify the local tissue fluid environment. Biological soft tissues exhibit mechanical properties that are influenced by fluid distribution, interstitial pressure, and connective tissue hydration. Consequently, changes in local tissue hydration and extracellular fluid balance may contribute to short-term alterations in elastographic stiffness following regional anesthesia [47,48,50].

Importantly, these physiologic effects differ fundamentally from mechanisms responsible for increased stiffness in chronic neuropathy. Whereas entrapment neuropathy and fibrosis are associated with persistent structural remodeling, stiffness changes after regional anesthesia may primarily represent reversible modulation of tissue mechanics.

### 4.3. Neural Unloading and Reduced External Compression

One of the most plausible explanations for reduced nerve stiffness after ACB is the concept of neural unloading. Peripheral nerves normally exist within a mechanically dynamic environment influenced by surrounding fascia, muscle contraction, vascular pulsation, and limb movement [27,33]. Changes in any of these factors may alter external compression forces applied to the nerve.

By mechanically expanding the adductor canal and separating fascial layers, perineural injectate may decrease localized compressive stress surrounding the saphenous nerve. Reduced external pressure may subsequently lower intraneural tension and increase nerve compliance. From an elastographic perspective, such unloading could appear as reduced shear wave velocity and lower stiffness measurements.

This concept may parallel observations from hydrodissection procedures used in entrapment neuropathy treatment. Ultrasound-guided hydrodissection has been shown to improve nerve mobility and reduce mechanical adhesion within compressed nerve tunnels [51]. Although ACB is not intended as a decompressive intervention, similar biomechanical mechanisms may occur transiently after injectate administration.

Another contributing factor may involve changes in neural excursion. Peripheral nerves normally glide relative to surrounding tissues during limb motion. Fascial expansion and decreased tissue adhesion after injection may increase nerve mobility and reduce mechanical resistance to deformation [27,51]. Enhanced neural compliance may further contribute to decreased stiffness measurements observed on SWE (Table 2).

The adductor canal may be particularly suitable for demonstrating such effects because it represents a relatively confined interfascial compartment with consistent sonographic anatomy. Small changes in local tissue pressure or fascial geometry may therefore produce measurable alterations in neural biomechanics.

### 4.4. Acute Physiologic Modulation Versus Chronic Structural Pathology

Most prior SWE studies have interpreted increased peripheral nerve stiffness as a marker of chronic pathology, including fibrosis, edema, and mechanical compression [8,9,11,23]. However, recent elastographic studies suggest that peripheral nerve stiffness is not a fixed mechanical property and may undergo rapid, reversible changes in response to alterations in neural loading, tissue tension, and local physiologic conditions. Preliminary evidence from regional anesthesia models further supports the possibility that nerve block procedures may transiently modify neural mechanical properties [14,26,35].

This distinction between chronic structural change and acute functional adaptation is important for interpreting elastographic findings. Elevated stiffness in chronic neuropathy likely reflects persistent connective tissue remodeling and impaired neural compliance. In contrast, reduced stiffness after ACB may represent transient alteration of tissue loading conditions rather than intrinsic structural change.

Consequently, peripheral nerve stiffness should be viewed as a context-dependent biomechanical parameter rather than a fixed indicator of pathology alone. Similar to vascular tone or compartmental pressure, stiffness may fluctuate dynamically in response to local physiologic conditions [9,26,33,35].

This evolving perspective expands the potential role of SWE in regional anesthesia research. Instead of serving solely as a diagnostic tool for neuropathy, elastography may provide quantitative insight into the biomechanical effects of nerve blocks, tissue decompression, injectate spread, and neural physiology in vivo.

Importantly, interpretation of decreased stiffness after regional anesthesia requires caution. Reduced stiffness should not automatically be assumed to represent beneficial or protective change, nor should increased stiffness necessarily indicate injury. Elastographic findings must be interpreted alongside clinical outcomes, neurologic examination, injection characteristics, and conventional ultrasonographic findings.

### 4.5. Implications for Future Research

The dynamic changes in peripheral nerve stiffness observed after regional anesthesia raise several important research questions. First, the temporal profile of stiffness changes remains unclear. Whether decreased stiffness persists only immediately after injection or continues throughout the duration of analgesia has not yet been established.

Second, the relationship between stiffness change and clinical block characteristics warrants investigation. It remains unknown whether greater stiffness reduction correlates with block success, analgesic quality, injectate spread, or patient outcomes. Quantitative elastographic assessment may eventually provide an objective biomarker for evaluating regional block dynamics.

Third, the safety implications of SWE require further study. Since excessive injection pressure and intraneural injection may produce mechanical injury, elastography could potentially assist in identifying abnormal tissue responses during block performance [13]. Continuous or serial monitoring of nerve stiffness may offer additional insight into early biomechanical changes preceding overt neurologic injury.

Finally, standardization of SWE methodology is essential before widespread clinical adoption. Probe orientation, limb position, timing of measurements, and ultrasound system variability all significantly influence elastographic results [52]. Establishment of standardized acquisition protocols will therefore be necessary for meaningful comparison across future studies.

Taken together, current evidence supports the concept that peripheral nerve stiffness is a dynamic and modifiable parameter influenced by regional anesthesia. The adductor canal provides a uniquely suitable experimental model for exploring these biomechanical interactions, and SWE may emerge as a valuable tool for quantitative assessment of peripheral nerve physiology during regional anesthesia practice.

## 5. Why Adductor Canal Block Is an Ideal SWE Model

### 5.1. Favorable Anatomical Characteristics for Elastographic Assessment

Among the various peripheral nerve block techniques used in regional anesthesia, adductor canal block (ACB) possesses several unique anatomical and technical characteristics that make it particularly suitable for shear wave elastography (SWE)-based investigation. The adductor canal contains the saphenous nerve within a relatively superficial and anatomically reproducible interfascial compartment, thereby facilitating consistent ultrasonographic visualization and serial stiffness measurement [29,53].

The saphenous nerve is typically identified adjacent to the femoral artery beneath the sartorius muscle at the mid-thigh level. Compared with deeper nerves such as the sciatic or lumbar plexus, the superficial location of the saphenous nerve minimizes signal attenuation and improves image quality during elastographic acquisition [10]. Because SWE reliability decreases with increasing tissue depth, the relatively shallow position of the saphenous nerve represents a major technical advantage [52,54].

In addition, the adductor canal exhibits consistent sonographic landmarks, including the sartorius muscle, femoral artery, and vastus medialis muscle. These reproducible anatomical relationships facilitate standardized probe placement and reduce interobserver variability. Standardization is particularly important in elastographic studies because stiffness measurements are highly sensitive to probe orientation, tissue anisotropy, and region-of-interest positioning [52].

Another important feature is the confined fascial architecture of the adductor canal. The vastoadductor membrane and surrounding muscular boundaries create a semi-enclosed compartment capable of transmitting pressure and mechanical forces after injectate administration [55]. This environment provides a favorable experimental setting for investigating how local anesthetic injection alters tissue biomechanics and peripheral nerve stiffness.

### 5.2. Suitability for Dynamic and Serial Measurements

ACB is especially advantageous for studying dynamic changes in nerve stiffness because repeated ultrasonographic examination can be performed relatively easily and reproducibly. The superficial location of the saphenous nerve permits serial SWE measurements before block performance, immediately after injection, and throughout the postoperative period without significant patient discomfort or technical difficulty.

Longitudinal assessment may be particularly valuable because peripheral nerve stiffness is likely to evolve over time following regional anesthesia [35]. Early changes may primarily reflect mechanical effects of injectate expansion, fascial separation, and hydrodissection [51]. In contrast, later alterations may be influenced by secondary physiologic processes, including changes in neural perfusion, tissue hydration, vascular dynamics, and local biomechanical conditions [14]. Understanding these temporal patterns may provide further insight into the mechanisms underlying elastographic changes after nerve block procedures.

Compared with deeper regional blocks, serial evaluation in ACB is less affected by patient positioning constraints or ultrasound accessibility limitations. Furthermore, ACB is commonly performed in awake or minimally sedated patients, allowing repeated assessments before block placement, immediately after injection, and during subsequent postoperative time points without major disruption to clinical workflow. This longitudinal accessibility is particularly advantageous for investigating temporal changes in nerve stiffness and distinguishing immediate mechanical effects of injectate expansion from delayed physiologic responses. Consequently, the adductor canal represents a practical and highly reproducible model for studying dynamic alterations in peripheral nerve biomechanics following regional anesthesia.

Continuous catheter-based ACB techniques further increase the value of the adductor canal as an elastographic research model. Continuous infusion permits investigation of prolonged tissue exposure to local anesthetics and enables evaluation of temporal stiffness changes over several hours or days [16]. Such longitudinal monitoring may improve understanding of the relationship between neural biomechanics, analgesic duration, and tissue recovery.

### 5.3. Relationship Between Fascial Mechanics and Injectate Spread

The adductor canal also provides a valuable model for investigating interactions between injectate spread and compartmental biomechanics. Unlike open anatomical regions with diffuse fluid distribution, the adductor canal confines injectate within a relatively narrow interfascial pathway [56]. Consequently, local anesthetic administration may produce measurable alterations in fascial tension, tissue compliance, and neural loading conditions.

Cadaveric and imaging studies have demonstrated that injectate within the adductor canal may spread proximally toward the femoral triangle or distally toward the popliteal region depending on injection site and volume [53]. This predictable yet variable spread pattern creates an opportunity to correlate elastographic changes with injectate distribution and compartmental expansion.

Perineural hydrodissection within the canal may decrease adhesion between the saphenous nerve and surrounding fascial structures, thereby modifying nerve mobility and mechanical stress [51]. These biomechanical alterations may be particularly detectable using SWE because elastography is sensitive to subtle changes in tissue tension and compliance.

**Figure 4:**
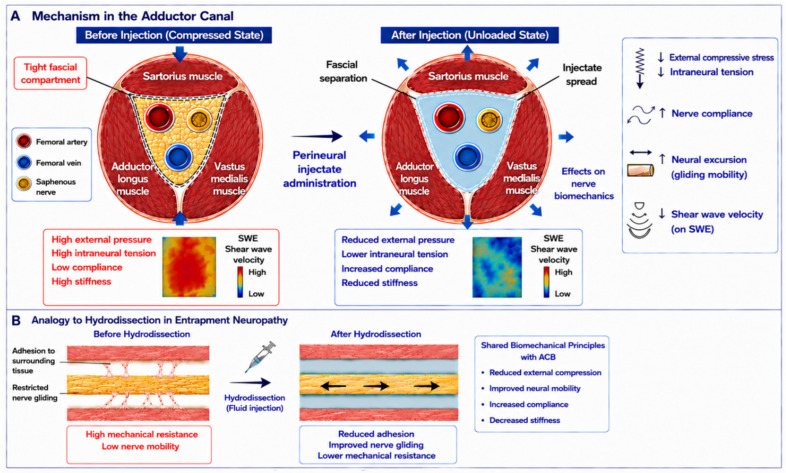
Hypothesized biomechanical effects of perineural injection on peripheral nerve stiffness. (**A**) Local anesthetic injection within the adductor canal produces fascial expansion and compartmental pressure redistribution, leading to reduced external mechanical constraint on the saphenous nerve. These changes may decrease intraneural tension, increase neural compliance, and ultimately reduce stiffness measurements obtained by shear wave elastography. (**B**) Hydrodissection-like effects of perineural injectate. Separation of interfascial planes decreases tissue adhesion and facilitates nerve excursion, thereby reducing mechanical resistance and enhancing neural mobility. Together, these mechanisms may explain the transient decrease in peripheral nerve stiffness observed after adductor canal block. Figure created using ChatGPT (https://chatgpt.com), accessed on 20 May 2026. Abbreviations: ACB, adductor canal block; SWE, shear wave elastography.

Furthermore, the canal’s fascial boundaries may amplify physiologic changes induced by local anesthetics. Sympathetic blockade-related vasodilation, tissue hydration changes, and reduction in muscular tension may collectively influence compartmental pressure within this confined space [14,46]. As a result, the adductor canal may serve as a physiologically responsive model for evaluating dynamic peripheral nerve mechanics (Figure 4).

### 5.4. Clinical Relevance of the Adductor Canal Block Model

Beyond its technical advantages, ACB possesses substantial clinical relevance that strengthens its value as an SWE research model. ACB has become one of the most widely used regional analgesic techniques for total knee arthroplasty and other knee surgeries because it provides effective sensory analgesia while relatively preserving quadriceps strength [1,3].

This widespread clinical adoption increases the translational significance of elastographic findings obtained in the adductor canal. Understanding how nerve stiffness changes after ACB may contribute not only to biomechanical knowledge but also to optimization of perioperative analgesic techniques.

Potential clinical applications include objective assessment of block dynamics, monitoring of injectate distribution, and early detection of abnormal tissue responses [52]. Quantitative stiffness analysis may eventually complement conventional ultrasonography by providing functional information regarding tissue compliance and neural physiology.

Additionally, the sensory-predominant nature of the saphenous nerve reduces confounding effects related to major motor blockade. Compared with femoral nerve block, ACB generally preserves ambulation and postoperative mobility, making postoperative serial examination more feasible in awake and cooperative patients [3].

Because the adductor canal is both anatomically accessible and clinically important, it represents an ideal intersection between experimental biomechanics and practical perioperative medicine. This combination distinguishes ACB from many other regional anesthesia techniques that may be technically less reproducible or clinically less amenable to repeated elastographic assessment.

### 5.5. ACB as a Translational Model for Peripheral Nerve Biomechanics

The emerging observation that saphenous nerve stiffness may decrease after ACB highlights the broader translational potential of this model. Traditionally, peripheral nerve stiffness has been interpreted primarily as a marker of chronic pathology such as fibrosis or compression neuropathy. However, dynamic stiffness reduction following regional anesthesia suggests that neural biomechanics may be acutely modifiable under physiologic conditions.

ACB therefore provides a unique in vivo experimental platform for studying how peripheral nerves respond to mechanical, vascular, and pharmacologic influences in real time. By integrating SWE with conventional ultrasonography, injection pressure monitoring, and clinical outcome assessment, future studies may clarify the relationship between tissue biomechanics and block physiology.

Importantly, the adductor canal model may also contribute to broader understanding of peripheral nerve mechanobiology. Insights gained from elastographic evaluation during ACB may ultimately have implications beyond regional anesthesia, including nerve entrapment syndromes, hydrodissection therapy, postoperative neural recovery, and rehabilitation medicine.

Taken together, the combination of favorable anatomy, reproducible imaging conditions, dynamic physiologic responsiveness, and high clinical relevance makes ACB one of the most promising models for investigating peripheral nerve stiffness using SWE.

## 6. Potential Clinical Applications

### 6.1. Quantitative Assessment of Regional Block Dynamics

One of the most promising clinical applications of shear wave elastography (SWE) in regional anesthesia is the quantitative assessment of block-related tissue dynamics. Conventional ultrasonography primarily provides qualitative visualization of needle placement and injectate spread, whereas SWE has the potential to provide objective biomechanical information regarding tissue response after local anesthetic administration [52].

Changes in peripheral nerve stiffness following regional anesthesia may reflect alterations in tissue compliance, compartmental pressure, neural mobility, and vascular dynamics. Quantitative monitoring of these changes could improve understanding of block physiology and potentially allow objective evaluation of block evolution over time.

Emerging observations of reduced saphenous nerve stiffness after adductor canal block (ACB) suggest that elastographic changes may correlate with physiologic neural modulation. If validated, SWE may serve as a functional imaging biomarker capable of detecting tissue responses that are not apparent on conventional B-mode imaging alone.

Furthermore, serial elastographic assessment may provide insight into temporal changes associated with onset and resolution of nerve block. Monitoring stiffness before injection, immediately after injection, and during postoperative recovery may improve understanding of dynamic interactions among injectate spread, tissue pressure, and neural biomechanics.

### 6.2. Objective Evaluation of Injectate Distribution and Hydrodissection

Perineural hydrodissection is increasingly recognized as an important mechanical consequence of regional anesthesia. Local anesthetic injection may separate fascial layers, reduce tissue adhesion, and alter external compression forces surrounding peripheral nerves [51]. Because SWE is highly sensitive to changes in tissue tension and compliance, elastography may provide an indirect quantitative assessment of hydrodissection effects.

In the adductor canal, the confined fascial anatomy may amplify biomechanical changes induced by injectate expansion. Consequently, elastographic alterations may reflect successful fascial plane separation or decompression of the saphenous nerve. Such information could potentially complement conventional ultrasonographic visualization of fluid spread.

Quantitative analysis of tissue stiffness may also improve comparison among different injection techniques, injectate volumes, or catheter infusion protocols. Future studies may determine whether specific patterns of stiffness reduction correlate with optimal injectate distribution or analgesic efficacy.

Additionally, SWE may help identify asymmetric or incomplete injectate spread. Since conventional ultrasonography sometimes fails to distinguish between superficial fluid accumulation and effective perineural spread, functional biomechanical assessment may provide supplementary information regarding the physiologic consequences of injection.

### 6.3. Early Detection of Adverse Neural Responses

Peripheral nerve injury remains a rare but important complication of regional anesthesia. Mechanical trauma, intraneural injection, excessive injection pressure, ischemia, and chemical neurotoxicity have all been implicated in block-related neurologic injury [4]. Current intraoperative monitoring strategies primarily rely on patient feedback, injection pressure monitoring, nerve stimulation, and conventional ultrasonography.

Because SWE quantitatively evaluates tissue stiffness, elastography may potentially assist in detecting abnormal biomechanical responses associated with neural injury. Excessive intraneural pressure or fascicular edema may alter elastographic properties before overt neurologic deficits become clinically apparent [12].

For example, abrupt focal stiffness elevation during injection could theoretically indicate abnormal tissue resistance, intrafascicular edema, or early mechanical injury. Conversely, physiologic stiffness reduction after uncomplicated ACB may represent normal tissue decompression and hydrodissection. Distinguishing these patterns may improve procedural safety in the future.

Although this concept remains speculative, integration of SWE with injection pressure monitoring and real-time ultrasonography could eventually contribute to multimodal safety assessment during regional anesthesia procedures.

### 6.4. Biomarker for Peripheral Nerve Physiology

Beyond procedural guidance, shear wave elastography may emerge as a valuable biomarker of peripheral nerve mechanical and physiologic status. Unlike conventional electrodiagnostic testing, SWE provides noninvasive, real-time information regarding tissue stiffness and biomechanical behavior without requiring electrical stimulation. In addition to structural visualization obtained with conventional ultrasonography, elastography offers quantitative assessment of tissue mechanical properties, potentially providing complementary insight into neural function, tissue compliance, and physiologic adaptation [52].

In perioperative medicine, nerve stiffness may reflect dynamic interactions among vascular tone, tissue hydration, neural loading conditions, and surrounding muscular activity [33,35]. Consequently, elastographic assessment could potentially provide insight into physiologic responses during surgery, postoperative recovery, and rehabilitation.

The observation that nerve stiffness may decrease after ACB suggests that SWE could capture transient functional modulation rather than irreversible structural pathology alone. This evolving perspective expands the conceptual role of elastography from static diagnostic imaging toward dynamic physiologic monitoring.

Future applications may include evaluation of nerve recovery after surgery, assessment of chronic pain interventions, monitoring of hydrodissection therapy, and investigation of postoperative neuropathic complications.

### 6.5. Research Applications in Regional Anesthesia

The adductor canal provides a uniquely favorable experimental environment for translational biomechanical research. Its superficial anatomy, reproducible sonographic landmarks, and widespread clinical use facilitate prospective investigation of nerve stiffness changes under controlled conditions [57].

Potential research applications include comparison of different local anesthetic agents, investigation of volume-dependent stiffness responses, assessment of continuous catheter infusion effects, and correlation of elastographic findings with analgesic outcomes.

In addition, integration of SWE with advanced imaging techniques such as contrast-enhanced ultrasonography, Doppler imaging, or magnetic resonance neurography may further clarify the relationship between tissue biomechanics and nerve physiology.

Although the saphenous nerve in the adductor canal provides an ideal model for SWE because of its superficial location and consistent sonographic visualization, the potential applications of elastography may extend beyond nerve-specific blocks. In field blocks (including interfascial plane blocks) or plexus blocks, such as the pectoral (PECS) block, transversus abdominis plane (TAP) block, erector spinae plane (ESP) block, fascia iliaca compartment block, cervical plexus block, or lumbar plexus block, individual nerves are often difficult or impossible to visualize directly. In these settings, SWE may provide complementary information by quantitatively assessing changes in tissue stiffness, fascial plane expansion, injectate distribution, or compartment biomechanics rather than nerve stiffness alone. Future studies should investigate whether elastographic assessment of surrounding tissues can serve as an indirect marker of successful injectate spread or block performance in these anatomically complex regional anesthesia techniques.

Taken together, SWE has the potential to become an important investigational and clinical tool in regional anesthesia, providing quantitative information that extends beyond conventional morphologic imaging.

## 7. Current Limitations and Challenges

### 7.1. Lack of Standardization

Despite increasing interest in peripheral nerve elastography, one of the major limitations of SWE is the absence of standardized acquisition protocols. Elastographic measurements are highly sensitive to technical variables including probe orientation, transducer pressure, limb position, region-of-interest (ROI) selection, and ultrasound system settings [34,52,54,58].

Peripheral nerves exhibit marked anisotropy because of their longitudinal fascicular structure [33]. Consequently, stiffness measurements may differ substantially between longitudinal and transverse imaging planes. Small variations in insonation angle may therefore produce significant differences in measured values [35,52].

Furthermore, studies evaluating peripheral nerve stiffness have employed highly heterogeneous methodologies, limiting direct comparison across investigations [58]. Variations in ultrasound equipment, vendor-specific software algorithms, acquisition protocols, region-of-interest selection, and reporting metrics may substantially influence measured stiffness values [52,59]. As a result, considerable inter-study variability exists, complicating the establishment of normative reference values and reducing the generalizability of published findings (Table 3).

Standardization will be particularly important if SWE is to be incorporated into clinical regional anesthesia practice. Future multicenter studies will require reproducible imaging protocols and consensus regarding acquisition methodology.

### 7.2. Interobserver and Interdevice Variability

Another important challenge is variability among operators and ultrasound systems. Although SWE is generally less operator-dependent than strain elastography, measurement reliability still depends heavily on technical expertise and imaging consistency [6,58].

Minimal transducer compression can artificially increase stiffness values, especially in superficial nerves such as the saphenous nerve [54]. Patient movement, vascular pulsation, and surrounding muscle contraction may additionally interfere with shear wave propagation [35].

Interdevice variability represents a major obstacle to broader clinical adoption. Different ultrasound manufacturers employ distinct elastographic algorithms and postprocessing techniques, which may produce non-comparable stiffness measurements even in identical tissues [59]. Consequently, stiffness values obtained using one system cannot necessarily be generalized across platforms.

These limitations complicate interpretation of peripheral nerve stiffness findings and may partially explain inconsistent results among published studies.

### 7.3. Limited Understanding of Biological Interpretation

Although increased stiffness is commonly associated with chronic neuropathy, the precise biologic meaning of elastographic changes remains incompletely understood. Peripheral nerve stiffness reflects the combined influence of connective tissue architecture, vascular perfusion, extracellular fluid distribution, intraneural pressure, and surrounding mechanical forces [33,47,48].

Consequently, identical stiffness changes may arise from different physiologic or pathologic mechanisms. For example, increased stiffness may result from fibrosis, edema, compression, or vascular congestion [8,9,23], whereas decreased stiffness may reflect hydrodissection, reduced tissue tension, altered perfusion, or increased compliance [33,46,51].

The emerging observation of decreased stiffness following ACB highlights the complexity of interpreting elastographic findings. Without concurrent physiologic or histologic correlation, distinguishing beneficial physiologic modulation from abnormal tissue change remains challenging.

Therefore, elastographic findings should currently be interpreted cautiously and in conjunction with clinical assessment and conventional imaging.

### 7.4. Limited Clinical Outcome Correlation

At present, relatively few studies have directly correlated elastographic findings with clinically meaningful outcomes in regional anesthesia [14]. It remains unclear whether changes in peripheral nerve stiffness predict block success, analgesic duration, neurologic recovery, or postoperative complications.

Similarly, the temporal relationship between stiffness changes and neural function has not yet been clearly established. Whether stiffness reduction immediately after ACB persists during analgesia or correlates with patient-reported pain outcomes remains uncertain.

Most available studies also involve small sample sizes and observational designs, limiting generalizability [52,58]. Prospective longitudinal studies will therefore be necessary to determine the true clinical significance of elastographic changes after peripheral nerve block.

### 7.5. Technical Challenges in the Perioperative Environment

Application of SWE during perioperative care presents additional practical limitations. Surgical positioning, sterile conditions, postoperative dressings, and patient discomfort may complicate repeated elastographic acquisition.

Furthermore, perioperative physiologic changes such as temperature variation, fluid administration, vasopressor use, and muscular relaxation may independently influence tissue stiffness. These confounding variables may complicate interpretation of elastographic findings obtained during anesthesia and surgery.

Despite these challenges, the superficial and accessible anatomy of the adductor canal may partially mitigate some technical limitations, reinforcing its value as a research model for peripheral nerve biomechanics.

## 8. Future Directions

### 8.1. Toward Quantitative Functional Imaging in Regional Anesthesia

The integration of SWE into regional anesthesia research reflects a broader shift toward quantitative functional imaging. Traditional ultrasonography primarily provides structural visualization, whereas elastography offers the possibility of evaluating tissue mechanics and physiologic response in real time.

Future studies may establish peripheral nerve stiffness as a dynamic biomarker reflecting neural loading conditions, compartmental pressure, vascular regulation, and tissue compliance. Such developments could significantly expand the role of ultrasound in perioperative medicine.

In particular, the observation that nerve stiffness may decrease after ACB suggests that elastography could provide insight into physiologic neural modulation rather than structural pathology alone. This concept may fundamentally alter how peripheral nerve biomechanics are interpreted in clinical practice.

### 8.2. Integration with Multimodal Monitoring

Future regional anesthesia research will likely involve integration of SWE with additional physiologic monitoring modalities. Combining elastography with injection pressure monitoring, Doppler ultrasonography, contrast-enhanced imaging, or magnetic resonance neurography may improve understanding of neural tissue response during block performance.

Simultaneous evaluation of tissue perfusion and stiffness may clarify the relationship between vascular dynamics and neural biomechanics. Similarly, integration with electrophysiologic assessment may help determine whether stiffness changes correlate with neural conduction or functional recovery.

Such multimodal approaches may improve differentiation between physiologic tissue modulation and early pathologic injury.

### 8.3. Longitudinal and Continuous Monitoring

Continuous catheter-based ACB techniques provide a unique opportunity for longitudinal investigation of peripheral nerve stiffness. Serial elastographic measurements obtained over hours or days may clarify the temporal evolution of tissue biomechanics during prolonged local anesthetic exposure.

Future studies should investigate whether stiffness changes correlate with analgesic duration, sensory blockade intensity, rehabilitation progress, or postoperative recovery. Dynamic monitoring may additionally reveal delayed tissue responses not detectable immediately after injection.

Wearable ultrasound technologies and automated imaging systems may further facilitate repeated perioperative elastographic assessment in the future.

### 8.4. Artificial Intelligence and Automated Analysis

Artificial intelligence (AI)-assisted ultrasound analysis may significantly improve the reproducibility and clinical applicability of peripheral nerve elastography. Automated nerve segmentation, motion correction, and standardized ROI selection could reduce operator dependency and improve measurement consistency.

Machine learning algorithms may additionally identify complex stiffness patterns associated with successful blockade, abnormal injection dynamics, or early neural injury. Integration of AI-based analysis with real-time ultrasonography could ultimately support procedural decision-making during regional anesthesia.

Although these technologies remain investigational, they represent an important future direction for quantitative ultrasound imaging.

### 8.5. Translational Implications Beyond Regional Anesthesia

The biomechanical insights obtained from ACB and SWE research may have implications extending beyond regional anesthesia alone. Dynamic assessment of peripheral nerve stiffness may contribute to broader understanding of nerve entrapment syndromes, postoperative neuropathy, hydrodissection therapy, rehabilitation medicine, and chronic pain disorders.

Furthermore, investigation of acute physiologic modulation may challenge the traditional assumption that peripheral nerve stiffness primarily reflects chronic structural pathology. Instead, stiffness may represent a continuously adaptive parameter influenced by mechanical, vascular, and neural factors.

The adductor canal model therefore provides not only a practical experimental platform but also a conceptual framework for future studies of peripheral nerve mechanobiology.

### 8.6. Future Perspective

As quantitative ultrasound technologies continue to evolve, SWE may eventually become an important adjunctive tool in regional anesthesia practice. Standardized elastographic assessment could potentially improve understanding of injectate dynamics, tissue compliance, and neural physiology during peripheral nerve block.

However, substantial work remains necessary before routine clinical implementation can be achieved. Large prospective studies, methodological standardization, and validation against clinical outcomes will be essential.

Nevertheless, current evidence suggests that peripheral nerve stiffness is a dynamic physiologic parameter rather than merely a static marker of neuropathy. The ability to visualize and quantify these biomechanical changes in vivo may open new directions for both perioperative medicine and peripheral nerve research.

## Figures and Tables

**Table 1:** Comparison of Peripheral Nerve Stiffness Characteristics in Normal and Pathologic Conditions.

Characteristic	Normal Peripheral Nerve	Entrapment Neuropathy	Metabolic Neuropathy (Diabetic Neuropathy)	Inflammatory Neuropathy
Typical SWE Findings	Moderate stiffness with homogeneous elastographic appearance	Increased stiffness	Increased stiffness	Variable, but generally increased stiffness
Primary Pathophysiology	Normal neural compliance and physiologic mobility	Chronic mechanical compression and impaired nerve gliding	Microvascular dysfunction, fibrosis, advanced glycation end-product accumulation	Inflammatory edema, cellular infiltration, and connective tissue remodeling
Intraneural Edema	Absent	Common	Common	Common
Fibrosis/Connective Tissue Remodeling	Minimal	Progressive in chronic stages	Prominent	May develop in chronic disease
Microvascular Changes	Normal perfusion	Venous congestion and ischemia	Microangiopathy and impaired perfusion	Increased vascular permeability and inflammation
Neural Mobility	Preserved	Reduced	Reduced	Variable
Stiffness Change Compared with Healthy Nerves	Reference baseline	Increased	Increased	Usually increased
Clinical Significance of SWE	Establishes normal reference values	Adjunctive diagnostic marker and severity assessment	Early detection and monitoring of neuropathy progression	Assessment of disease activity and structural involvement
Potential Reversibility	Dynamic physiologic variation	Limited, depending on chronicity	Progressive but potentially modifiable	Variable depending on treatment response

**Table 2:** Proposed Mechanisms of Decreased Saphenous Nerve Stiffness After Regional Anesthesia.

Mechanism	Proposed Effect
Hydrodissection	↓ external compression
Fascial expansion	↓ tissue tension
Neural unloading	↑ compliance
Sympathetic blockade	↑ blood flow
Altered intraneural pressure	↓ stiffness
Muscle tone reduction	possible minor contribution

Symbols: ↑ indicates an increase; ↓ indicates a decrease.

**Table 3:** Factors Influencing Peripheral Nerve Shear Wave Elastography Measurements.

Factor	Potential Effect on SWE Measurement
Probe pressure	Artificial increase in stiffness
Insonation angle	Measurement variability due to anisotropy
Probe orientation (longitudinal vs. transverse)	Different stiffness values
Limb position	Changes in neural tension and stiffness
Muscle contraction	Increased external tissue tension
ROI selection	Variability in measured values
Tissue depth	Reduced signal quality
Vascular pulsation	Motion artifact
Ultrasound system/vendor	Interdevice variability
Operator experience	Reproducibility differences

## Data Availability

No new data were generated during the preparation of this review. Therefore, data sharing is not applicable.
